# Interstitial pneumonitis following the implantation of a paclitaxel-eluting stent in a patient with peripheral arterial disease

**DOI:** 10.1016/j.jvscit.2025.102096

**Published:** 2025-12-15

**Authors:** Makoto Haga, Yuko Iwata, Jun Nitta, Junetsu Akasaka

**Affiliations:** a Department of Cardiovascular Surgery, Tokyo Medical University Hachioji Medical Center, Tokyo, Japan; b Department of Respiratory Medicine, Tokyo Medical University Hachioji Medical Center, Tokyo, Japan

**Keywords:** Paclitaxel-eluting stent, Interstitial pneumonitis, Peripheral artery disease

## Abstract

We report a case of interstitial pneumonitis after paclitaxel-eluting stent implantation for peripheral arterial disease in a 74-year-old man. Five weeks after the procedure, the patient developed dyspnea and bilateral ground-glass opacities, with a paclitaxel dose (1169 μg) exceeding the recommended limit (1034 μg). Hypersensitivity to paclitaxel likely caused interstitial pneumonitis, confirmed by elevated Krebs von den Lungen-6 levels and exclusion of other etiologies. Corticosteroid therapy improved symptoms. Clinicians should monitor for rare but serious paclitaxel-induced pulmonary hypersensitivity in patients with peripheral arterial disease.

Paclitaxel-eluting devices (PESs) are widely used by vascular specialists worldwide to treat peripheral arterial disease (PAD). Previous studies suggest that paclitaxel-related drug eluting stents (DESs) and drug-coated balloons (DCBs) are superior to plain balloon angioplasty in reducing restenosis rates.^1^^,^^2^

Reports in the coronary literature have described interstitial pneumonitis (IP) after DES implantation.^3^, ^4^, ^5^, ^6^ Previous studies indicate that paclitaxel-induced IP occurs in approximately 1% of patients.^7^^,^^8^ Several cases of IP have also been reported in patients with breast and lung cancer treated with paclitaxel.^9^, ^10^, ^11^ However, no cases have been described after paclitaxel device use in peripheral arteries. We present a case of IP after PES angioplasty in a patient with PAD. Informed consent was obtained from the patient for the case report and images.

## Case report

**Fig 1:**
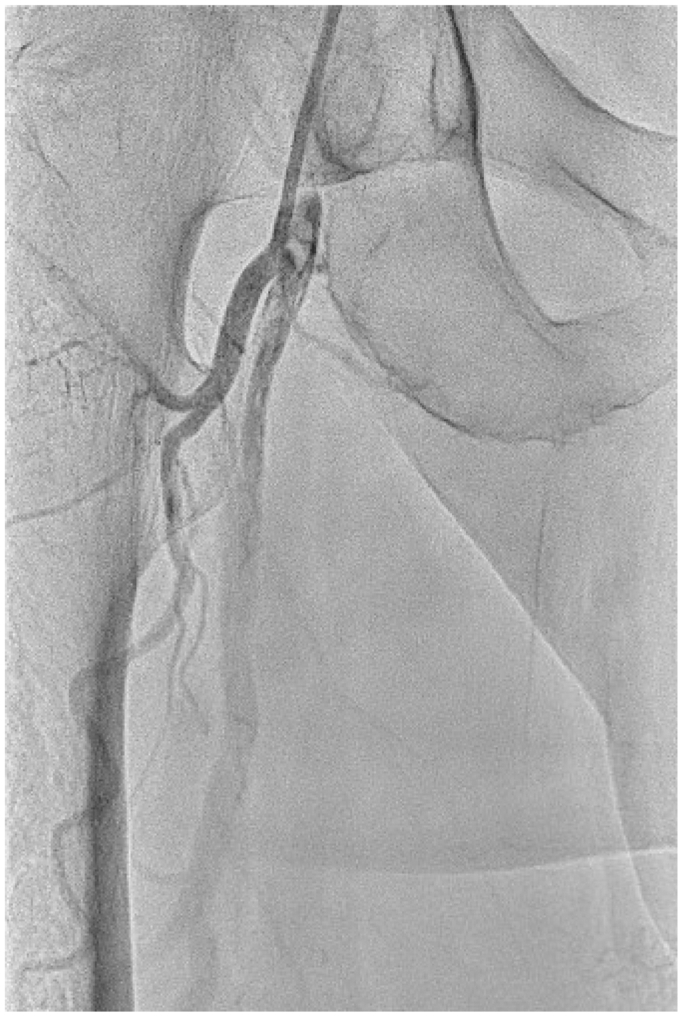
Initial angiography demonstrating chronic total occlusion of the right superficial femoral artery (SFA).

**Fig 2:**
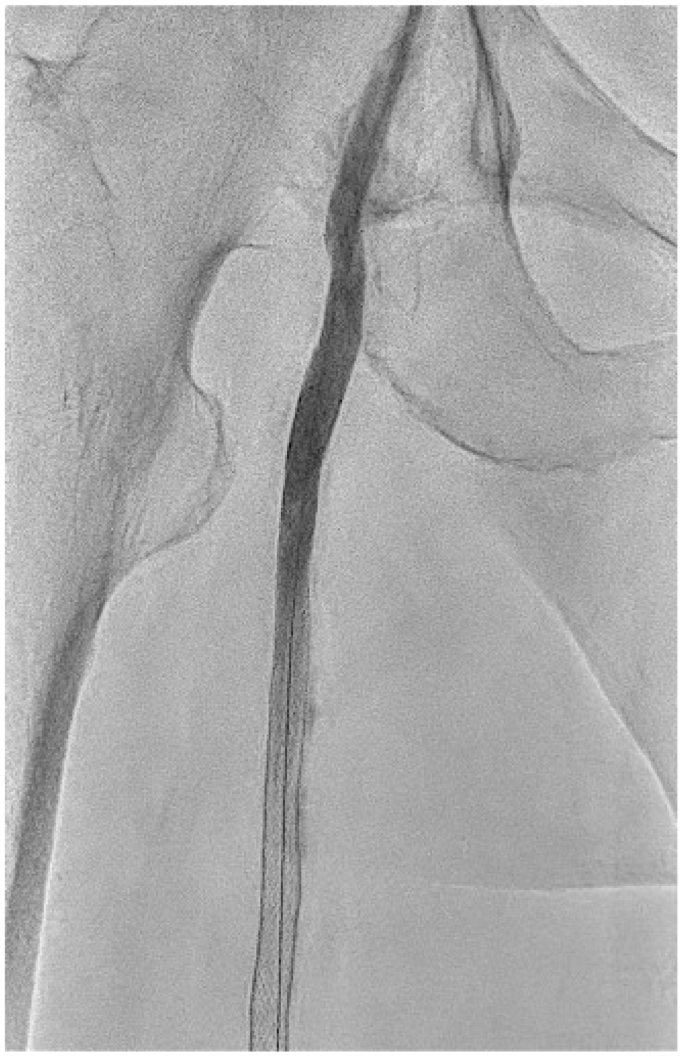
Final angiography after implantation of three paclitaxel-eluting stents (total paclitaxel dose: 1169 μg) in the right superficial femoral artery (SFA), showing successful revascularization. No pseudoaneurysm or dissection was identified.

A 74-year-old man with PAD underwent endovascular revascularization of the right superficial femoral artery (SFA) using a PES (Eluvia; Boston Scientific) in September 2023 at a local hospital. The initial angiography showed chronic total occlusion in the right SFA (Fig 1). Three PESs (two 6 × 150 mm and one 7 × 40 mm Eluvia) were implanted in the right SFA, delivering a total paclitaxel dose of 1169 μg (Fig 2). No pseudoaneurysm or dissection was identified on the initial or final angiograms (Figs 1 and 2). Aspirin (100 mg/d) and clopidogrel (75 mg/d) were started postoperatively.

**Fig 3:**
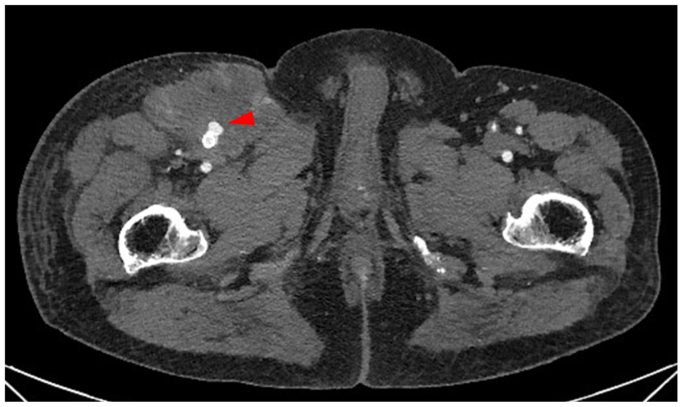
Computed tomography (CT) angiography revealing a pseudoaneurysm (*arrow*) at the proximal segment of the right superficial femoral artery (SFA).

**Fig 4:**
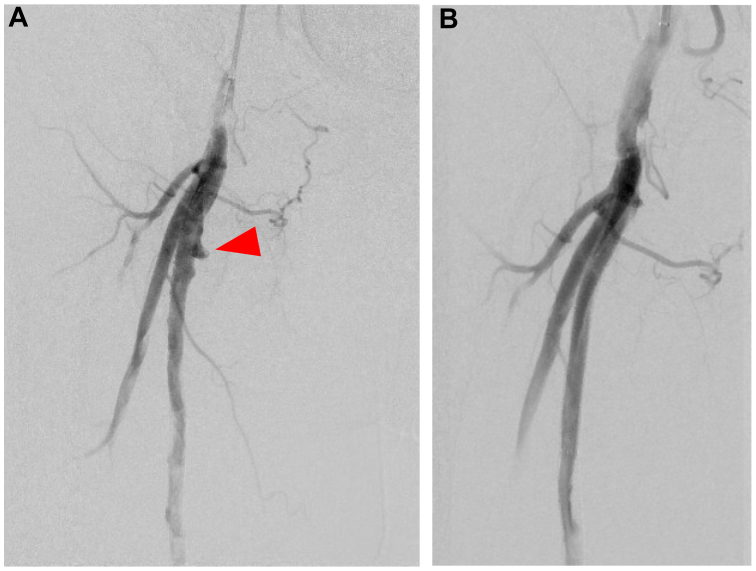
**A,** Preoperative angiography confirming the presence of a pseudoaneurysm (*arrow*) in the proximal right superficial femoral artery (SFA). **B,** Postoperative angiography confirmed complete coverage of the pseudoaneurysm by the stent graft and absence of residual contrast extravasation in the right SFA.

A few days later, the patient developed right groin pain and swelling. Computed tomography (CT) angiography at the referring hospital revealed a pseudoaneurysm in the right proximal SFA (Fig 3). The patient was transferred to our hospital, where emergency revascularization was performed. Preoperative angiography confirmed a pseudoaneurysm in the right proximal SFA (Fig 4, *A*); therefore, a stent graft (6 × 100 mm Viabahn; GORE) was implanted at the pseudoaneurysm site. Postoperative angiography confirmed no contrast leakage (Fig 4, *B*). The patient's symptoms improved, and follow-up CT angiography showed no extravasation. He was discharged 5 days later.

**Fig 5:**
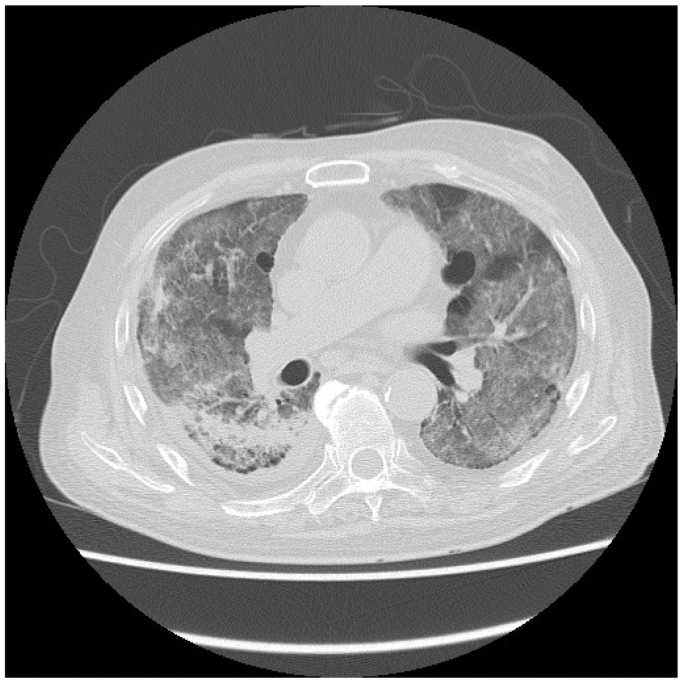
Chest computed tomography (CT) showing bilateral ground-glass opacities, predominantly in the peripheral and lower lung zones.

**Fig 6:**
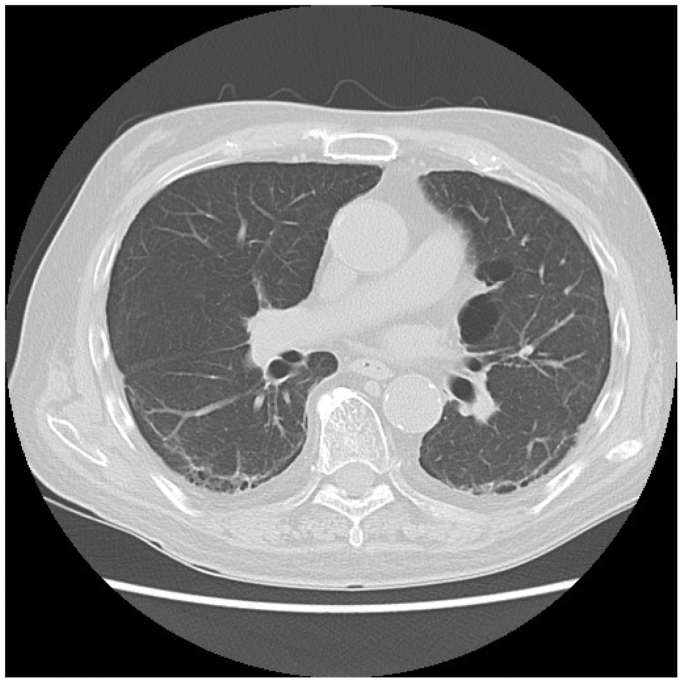
Follow-up chest computed tomography (CT) demonstrating gradual resolution of the previously noted bilateral ground-glass opacities.

Five weeks after the initial procedure, the patient presented with dyspnea, low-grade fever, and hypoxemia. Physical examination revealed bilateral fine crackles, and chest CT showed bilateral ground-glass opacities (Fig 5). Microbiological cultures and serologic testing excluded infectious and autoimmune causes. Krebs von den Lungen-6 protein was elevated (602 U/mL). The cumulative paclitaxel dose (1169 μg) exceeded the recommended upper limit (1034 μg). Based on clinical, radiological, and laboratory findings, a diagnosis of IP due to PES implantation was made. The duration of oral corticosteroid therapy (typically prednisolone) for IP generally follows established guidelines: an initial high-dose phase of 1 to 2 mg/kg/d for 2 to 4 weeks, followed by a gradual taper over 2 to 6 months. Prednisolone therapy (methylprednisolone 40 mg/d) was started. However, the patient's respiratory symptoms worsened within a week, necessitating a switch to high-dose corticosteroid pulse therapy. Over the next 2 months, the patient's symptoms and radiological findings (Fig 6) gradually improved, allowing the steroid dose to be tapered to oral prednisolone 12.5 mg/d. Low-dose maintenance therapy was considered only in cases of chronic progressive fibrotic disease or connective tissue disease-associated interstitial lung disease with persistent activity. However, prednisolone was discontinued at 6 months. No recurrence occurred, and the patient was discharged. The patient was followed for 15 months and is doing well.

## Discussion

Paclitaxel-related DESs and DCBs are established treatments for PAD. However, IP after PES implantation is rare and has been reported only in coronary interventions.^3^, ^4^, ^5^, ^6^ The timeframe from the start of paclitaxel treatment to the diagnosis of pneumonitis varies widely, influenced by factors such as treatment schedule (eg, weekly vs dose-dense), formulation (conventional paclitaxel vs nab-paclitaxel), combination therapies (eg, with radiation or gemcitabine), and patient-specific characteristics.^12^ For nab-paclitaxel in lung cancer, the median time to pneumonitis onset was 76 days.^12^ In early-stage breast cancer, the median time from the first paclitaxel infusion to pneumonitis development was 15 days (range: 2-20 days).^9^ The time from coronary DES placement to the diagnosis of IP varies widely, ranging from 3 days to 2 months across reported cases.^3^, ^4^, ^5^, ^6^ A duration of 5 weeks for IP diagnosis, as seen in this case, falls within this range and represents a plausible timeframe.

Possible mechanisms of IP include drug toxicity, autoimmune processes, infection, hypersensitivity, allergic responses, and radiation exposure.^4^^,^^5^^,^^13^ The primary cause of pneumonitis after DES implantation is a hypersensitivity reaction to the stent's eluted drug (eg, paclitaxel, everolimus, or sirolimus) caused by the polymer coating controlling drug release. The Eluvia stent, used in the SFA, uses a polymer-based coating (Sustend) that sustains paclitaxel release for over 1 year, aligning with the restenosis process, which typically occurs within months to a year. The polymer matrix may trigger delayed hypersensitivity, contributing to lung inflammation and IP. Drug-induced toxicity may represent a secondary etiology of IP. Although ticlopidine and certain other antiplatelet agents have rarely been implicated in drug-induced IP, no convincing reports associate aspirin with this condition.^14^ In this case, the delivered paclitaxel dose from the Eluvia drug-eluting stent (1169 μg) exceeded the labeled warning limit of 1034 μg total paclitaxel exposure, despite the dose density (0.167 μg/mm^2^) being within the approved specification and no predefined systemic limit existing for multiple stents or procedures. Pharmacokinetic data from the Eluvia drug-eluting stent demonstrate minimal systemic paclitaxel exposure, with plasma concentrations remaining below 1.0 ng/mL in nearly all patients and a maximum observed value of 1.60 ng/mL in only one patient at 10 minutes after implantation.^15^ These levels are transient and orders of magnitude lower than those used in oncologic therapy. Given the clear temporal association with stent implantation, the presence of hypersensitivity reaction, drug-induced toxicity, the lack of association with concomitant antiplatelet therapy, and the absence of alternative etiologies, paclitaxel emerges as the primary causative agent of IP in this patient.

Beyond pneumonitis, no pseudoaneurysm or dissection was identified on the initial or final angiograms during the first procedure. The pseudoaneurysm most likely formed gradually after angioplasty, possibly due to a subtle fracture in the calcified vessel wall or paclitaxel-induced vessel injury. Although a direct causal relationship with paclitaxel (as opposed to the angioplasty procedure itself) cannot be conclusively established, late aneurysm formation has been reported as a potential risk with paclitaxel-coated devices.^16^, ^17^, ^18^, ^19^, ^20^, ^21^, ^22^, ^23^ The primary reason for aneurysm formation after the implantation of DES, such as those used in coronary or peripheral arteries, is hypersensitivity-induced vascular inflammation and subsequent vessel wall remodeling. The eluted drug or polymer can trigger a local inflammatory response, characterized by eosinophilic infiltration or chronic inflammation, which weakens the vessel wall. This is supported by histopathological studies showing inflammatory cell infiltration in coronary artery aneurysms after DES implantation.^24^^,^^25^ The antiproliferative drugs in DES inhibit smooth muscle cell proliferation, which can delay endothelial healing and lead to medial wall thinning or necrosis, increasing the risk of aneurysm formation. Taken together, these findings underscore that both pulmonary and vascular hypersensitivities share a likely drug-mediated mechanism.

Although DESs and DCBs are superior to plain balloon angioplasty in terms of patency and restenosis rates,^1^^,^^2^ a recent meta-analysis demonstrated that there was no significant difference in the rate of ipsilateral major amputation and all-cause mortality between using paclitaxel-coated or uncoated devices.^26^ Given the potential risks of life-threatening IP and aneurysmal degeneration, the indiscriminate use of paclitaxel-coated technologies in PAD should be carefully reconsidered.

## Conclusions

PESs remain an effective option for reducing restenosis in PAD, but clinicians should remain vigilant for rare yet serious adverse events such as IP. Further research is required to better define the dose-response relationship, identify patient-specific risk factors, and clarify the underlying mechanisms of paclitaxel-induced pulmonary hypersensitivity.

## Funding

None.

## Disclosures

None.
